# Characterizing the soil microbiome and quantifying antibiotic resistance gene dynamics in agricultural soil following swine CAFO manure application

**DOI:** 10.1371/journal.pone.0220770

**Published:** 2019-08-19

**Authors:** Edward Lopatto, Jinlyung Choi, Alfredo Colina, Lanying Ma, Adina Howe, Shannon Hinsa-Leasure

**Affiliations:** 1 Department of Biology, Grinnell College, Grinnell, Iowa, United States of America; 2 Department of Agricultural and Biosystems Engineering, Iowa State University, Ames, Iowa, United States of America; Academia Sinica, TAIWAN

## Abstract

As agriculture industrializes, concentrated animal feeding operations (CAFOs) are becoming more common. Feces from CAFOs is often used as fertilizer on fields. However, little is known about the effects manure has on the soil microbiome, which is an important aspect of soil health and fertility. In addition, due to the subtherapeutic levels of antibiotics necessary to keep the animals healthy, CAFO manure has elevated levels of antibiotic resistant bacteria. Using 16s rRNA high-throughput sequencing and qPCR, this study sought to determine the impact of swine CAFO manure application on both the soil microbiome and abundance of select antibiotic resistance genes (ARGs) and mobile element genes (*erm(B)*, *erm(C)*, *sul1*, *str(B)*, *intI1*, IncW *repA*) in agricultural soil over the fall and spring seasons. We found the manure community to be distinct from the soil community, with a majority of bacteria belonging to Bacteroidetes and Firmicutes. The soil samples had more diverse communities dominated by Acidobacteria, Actinobacteria, Proteobacteria, Verrucomicrobia, and unclassified bacteria. We observed significant differences in the soil microbiome between all time points, except between the spring samples. However, by tracking manure associated taxa, we found the addition of the manure microbiome to be a minor driver of the shift. Of the measured genes, manure application only significantly increased the abundance of *erm(B)* and *erm(C)* which remained elevated in the spring. These results suggest bacteria in the manure do not survive well in soil and that ARG dynamics in soil following manure application vary by resistance gene.

## Introduction

In the past two decades, agriculture has become more industrialized and has shifted toward fewer but larger farm operations. Swine production is no exception to this trend; in the US, nearly three-quarters of swine are grown in concentrated animal feeding operations (CAFOs) containing over 5,000 pigs each [1]. Manure from swine CAFOs is often used as an organic fertilizer on fields to improve soil quality. Organic fertilizer provides nitrogen enrichment, increases soil organic matter, and is often thought to be a better alternative than synthetic fertilizer [2, 3]. The manure microbiome can influence the soil microbiome through direct competition and transfer of antibiotic resistance genes (ARGs) [4]. However, our knowledge of these processes is limited.

The soil microbiome has been linked to overall soil quality because it is involved in nutrient cycling, helps maintain soil water content, and influences soil acidity [5–8]. Manure application can impact the soil microbiome by increasing available nutrients or by introducing the manure microbiome [9, 10]. However, it is unclear how much of a role the introduction of the manure microbiome plays in altering the soil microbiome as members of the manure microbiome may not compete well in the soil environment [11, 12]. While previous studies have found that organic manure application significantly alters the soil microbiome, we still do not fully understand the extent to which manure application shifts the soil microbiome or the duration of the shift [9, 13, 14].

Manure from CAFOs has also been shown to be a reservoir of antibiotic resistant bacteria due to the use of subtherapeutic levels of antibiotics in the feed [15–19]. Because of high levels of antibiotic resistant bacteria in manure, manure application has been shown to significantly increase the abundance of ARGs in soil [20–24]. From the soil, ARGs can be dispersed into surrounding waterways via runoff and drainage [25–27]. As ARGs may be transferred to pathogenic bacteria in the environment through horizontal gene transfer, agricultural dissemination of ARGs into the environment may perpetuate the current increase of antibiotic resistance, which is a public health threat as the number of antibiotics available becomes more limited [15, 28, 29].

In the present study, we investigated the impact of swine CAFO manure fertilization on both the soil microbiome and the abundance of antibiotic resistance and mobile element genes over the fall and spring seasons. The effect of manure fertilization on the soil microbial community was explored by using 16S rRNA gene high-throughput sequencing. qPCR was employed to determine the relative abundances and dynamics of select antibiotic resistance and mobile element target genes (*erm(B)*, *erm(C)*, *sul1*, *str(B)*, *intI1*, IncW *repA*) from soil samples over the fall and spring season. These genes were chosen because they represent resistance to a variety of antibiotics commonly used in the swine industry, including the CAFO in this study, and because they overlap ARGs investigated in several studies, most specifically with those used by Marti et al., allowing for us to begin building comparisons across environments [23]. The present study found that manure application may not be a significant factor in altering the soil community over a five-month period but does affect the gene abundance of select resistance genes.

## Materials and methods

### Study site

Manure, manure line, and field soil samples were taken from a swine CAFO farm located near Grinnell, IA (41.5896, -92.7569). Permission was granted by the land owner for sample collection. The CAFO houses approximately 6,500 hogs. Manure was collected in a pit below the CAFO for a year before application. The year before sampling the following antibiotics were dispensed in the feed according to a feeding regimen for growing swine, which included: tiamulin, chlortetracycline, sulfamethazine, penicillin, lincomycin, and tylosin phosphate. When infections arose in the barn, swine were additionally treated with penicillin and cycline, separately supplied in water, and 60 swine had Indoflex shots to treat illness.

The agricultural field sampled is a flat section of land at the bottom of the field that slopes up to the farm buildings. The field was split into two sites with each site centered around a tile line that empties into the stream positioned next to the field. The field is primarily silt loam soil (https://websoilsurvey.sc.egov.usda.gov/) [30]. Soil samples were collected before manure application on November 8, 2016. Manure was applied on the corn field for the 2017 growing season on November 12, 2016 at a rate of 4,127 gallons per acre, and the manure was sampled at this time. Manure was injected into knife lines running parallel through the field, and after 2–3 weeks the entire field is tilled. The samples collected just after application, on November 15, 2016, are from the injection lines referred to as “manure lines” and from soil in between the injection lines “soil”. After tilling samples were collected from the mixed soil on February 17, 2017 and March 22, 2017. There were no samples available on this site that had not been impacted by animal manure.

### Sample collection

For field soil and manure line samples, soil cores were taken at random locations within each site using a T-sampler sterilized with 70% ethanol between samplings. Samples were contained in a sterile bag and mixed by hand to homogenize the sample. Samples were then transported to the lab and refrigerated within one hour of collection and stored frozen at -20°C. Data from the two sites were combined for analysis for each respective sample type and date.

### DNA extraction

For 16S community analysis, DNA was extracted from 250 mg of soil and 250 μl of manure samples using the MagAttract Powersoil DNA EP Kit (384) (Qiagen, Germantown, MD) according to the manufacturer’s instructions. DNA was extracted from ten manure samples, twelve manure line samples from each site, and twelve soil samples from each site for every sampling date.

For qPCR, DNA was extracted from 250 mg of soil and 250 μl of manure samples using the DNeasy PowerSoil Kit (Qiagen, Germantown, MD) using the manufacturer’s instructions with one modification: the samples were placed on a Mini-Beadbeater (Biospec Products, Bartlesville, OK) for 140 seconds to homogenize. In total, DNA was extracted from ten manure samples, ten manure line samples from each site, and 10 soil samples from each site for every sampling date.

### 16S rRNA community analysis

The V4 region of the 16S rRNA gene was amplified using the Earth Microbiome Project primers (F: 5’GTGYCAGCMGCCGCGGTAA3’, R: 5’GGACTACNVGGGTWTCTAAT3’; fwd-barcoded: 515FB-806RB) and standard protocol [31]. Samples were indexed and sequenced using Illumina MiSeq (Illumina, San Diego, CA) producing 250-bp paired-end reads. Sequences were processed with MOTHUR (version 1.39.5) [32] according to standard operating procedure (http://www.mothur.org/wiki/MiSeq_SOP) [33]. Briefly, paired sequences were joined, primers were trimmed, and sequences were screened. The SILVA 16S rRNA sequence database (Release 132) was used for reference alignment and the RDP 16S rRNA reference (http://rdp.cme.msu.edu) for taxonomic classification [34, 35]. The VSEARCH algorithm in MOTHUR was used to filter chimeras. The OptiClust algorithm in MOTHUR was used to cluster processes sequences into operational taxonomic units (OTUs) using a 97% sequence similarity cutoff [36]. For the purposes of this study, only sequences associated with bacteria were used for analysis.

Statistical analysis and data visualization were carried out in R (version 3.5.0) (https://www.r-project.org/). The package phyloseq (version 1.24.2) was used for rarefying and data cleaning [37]. The packages ape (version 5.1) [38], dplyr (version 0.7.6) [39], and reshape2 (version 0.8.7) [40] functions were used in data transformations. Both alpha diversity indices and relative phyla abundance were produced in phyloseq and analyzed using ANOVA from the package vegan (version 2.5–2) [41]. NMDS ordination was produced and analyzed using the ADONIS function, which is equivalent to a PERMANOVA, using the vegan package. DEseq2 was used to calculate differential abundance of OTUs after singletons were removed (version 1.20.0) [42]. The package ggplot2 (version 3.0.0) [43] was used for visualization along with RColorBrewer (version 1.1–2) [44].

### qPCR for gene targets

Quantitative Real-Time PCR amplification was performed using an Applied Biosystems StepOnePlus Real-Time PCR System (ThermoFisher, Waltham, MA). The primers used in this study are described in Table 1, and were obtained from Integrated DNA Technologies in Coralville, IA.

**Table 1:** qPCR primers and probes used in this study.

Name	Sequence (5'→3')^a^	Annealing Temp (C)	Final primer concn (nM)	Target	Reference
**Universal bacteria**		59	300	*rrnS* gene	[45]
BACT1369F	CGGTGAATACGTTCYCGG				
PROK1492R	GGWTACCTTGTTACGACTT				
TM1389F	HEX-CTTGTACACACCGCCCGTC-BHQ1				
***erm*(B)**		65	200	Erythromycin resistance gene locus B	[46]
ermB-F	AAAACTTACCCGCCATACCA				
ermB-R	TTTGGCGTGTTTCATTGCTT				
***erm(C)***		62	200	Erythromycin resistance gene locus C	[47]
ermC-F	AATCGTGGAATACGGGTTTGC				
ermC-R	CGTCAATTCCTGCATGTTTTAAGG				
***sul1***		64	200	Sulfamethazine resistance gene 1	[23]
sul1-F	GACTGCAGGCTGGTGGTTAT				
sul1-R	GAAGAACCGCACAATCTCGT				
***str*(B)**		61	300	Streptomycin phosphotransferase B	[48]
strB-F	ATCGCTTTGCAGCTTTGTTT				
strB-R	ATGATGCAGATCGCCATGTA				
strB-P	HEX-ATGCCTCGGAACTGCGT-BHQ1				
***intI1***		62	200	Integrase class 1	[49]
Int1F2	TCGTGCGTCGCCATCACA				
Int1R2	GCTTGTTCTACGGCACGTTTGA				
**IncW *repA***		61	300	*repA* gene from plasmid incompatibility group W	[23]
IncW-F	GGCCATCGTATCAACGAGAT				
IncW-R	ATTGGTGCGCTCAAAGTAGC				
IncW-P	HEX-AGCTGGCTTAGTCGGCTACA-BHQ1				

^a^ HEX, 2’, 4’, 5’, 7’-tetrachloro-6-carboxy-4,7-dichlorofluorescein succinimidyl ester; BHQ1, black hole quencher 1.

Plasmids containing *erm(C)* target DNA fragment were provided by the Howe Lab at Iowa State University [24]. Plasmids containing all other target DNA fragments for every primer were provided by Marti et. al [23]. Plasmids were transformed into *Escherichia coli* One Shot TOP10 Cells (Thermofisher, Waltham, MA) using the manufacturer’s instructions and were extracted using the Wizard Plus SV Minipreps DNA Purification System (Promega, Madison, WI) following the manufacturer’s instructions. Plasmid copy number was calculated using the NanoDrop One Microvolume UV-Vis Spectrophotometer (ThermoFisher, Waltham, MA). The cultivated plasmids were used for standard curves consisting of 10-fold samples.

Each reaction was prepared using 12.5 μl of Takyon ROX SYBR serial dilutions that spanned the range of the target gene amplification from environmental MasterMix blue dTTP (Eurogentec, Fremont, CA) for SYBR green PCR and PrimeTime Gene Expression Master Mix (Integrated DNA Technologies, Coralville, IA) for TaqMan PCR. Two microliters of template DNA and deionized water were added to reach a final volume of 25 μl. Every sample, including a no template DNA control of DNA free water, was run in triplicate. A melting curve afterwards was used to check the purity of the SYBR green assay qPCR product.

### Gene abundance analysis

The quantification thresholds and cycle were determined using the StepOne Software (Version 2.0.2) (Applied Biosystems). Standard curves were generated using linear regression analysis of the quantification cycle versus the amount of template DNA. A regression goodness of fit (r^2^) of above 0.9 was needed for the qPCR run to be used in analysis. Amplification efficiency was calculated from the linear regression as described previously [50]. Amplification efficiency between 90% and 110% was needed in order for the qPCR run to be used in analysis. Limit of detection (LOD) and limit of quantification (LOQ) were determined by serial dilutions of known plasmid amounts. The LOD was the lowest dilution that was distinguishable from the no template control. The LOQ was the lowest dilution that stayed within one standard deviation of the linear regression line and was distinguishable from the no template control. Gene target copy number was calculated using the standard curve and each gene abundance is expressed as a ratio of targeted gene copy per total rrnS gene copy in the reaction.

GraphPad Prism 7 (GraphPad Software, Inc.) was used for statistical analysis and the results were visualized in R. Gene abundances were compared using Kruskal-Wallis test with Dunn’s test for multiple comparisons using Luby et al.’s method to compensate for LOD and LOQ abundances [24]. Briefly, samples with gene abundance below the specified LOQ and above the LOD were assigned the average of the LOQ and LOD for analysis. Sample abundances below the LOD were assigned a value of one for analysis. This does not alter statistical significance because Kruskal-Wallis is a non-parametric rank based test.

## Results

### Sample collection

Manure, soil, and manure line samples were obtained from a swine CAFO located near Grinnell, IA. Ten manure samples were taken directly from the lagoon beneath the swine CAFO at the same time of its application onto the field, November 12, 2016. Soil samples were obtained from two sites from a commercial agricultural field. Data from the two sites were combined for analysis for each respective sample type and date. Twelve soil samples were taken from each site at four time points: fall pre-manure application (November 8, 2016), fall post-manure application (November 15, 2016), spring time 1 (February 17, 2017) and spring time 2 (March 22, 2017).

### General description of DNA sequences

There was an average of 28,129 sequences per an individual soil or manure sample. Sequences were rarefied to 19,239 reads for analysis (the lowest sampling depth of this experiment that provided adequate taxa coverage), and samples below the rarefying depth were discarded. Rarefaction curves suggest that the rarefying depth covered the dominant taxa. After rarefying, 125 out of 130 samples remained, representing a total of 2,404,875 reads, 25,514 Operational Taxonomic Units (OTUs) at 97% similarity, and 33 phyla. The sequences have been deposited in NCBI repository with the accession no. SRP158016.

### Characterization of bacterial communities

**Fig 1:**
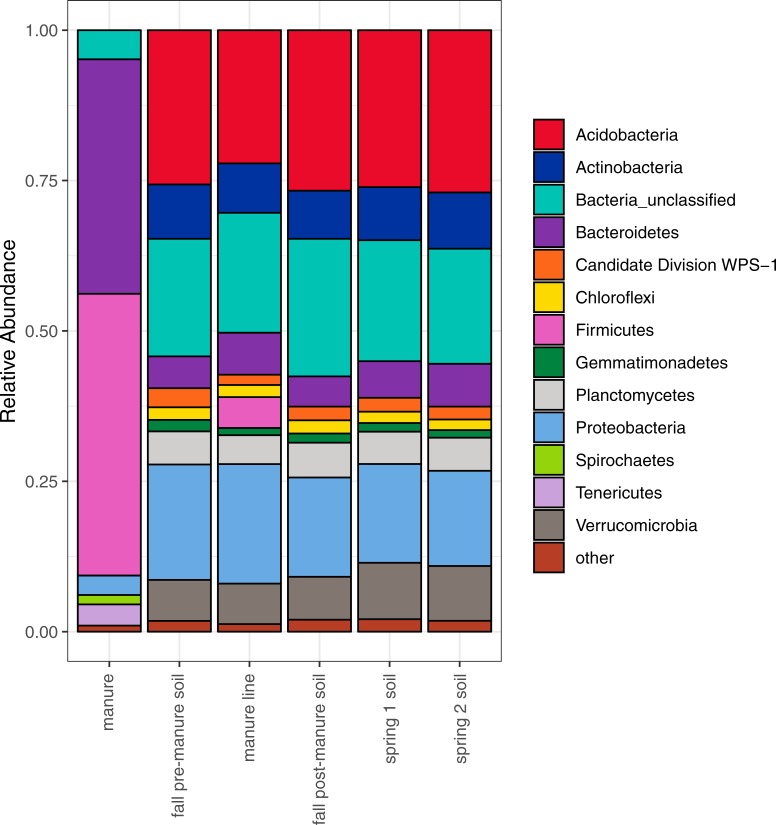
Average relative abundances of phyla among sample types. Included in “Other” are phyla that individually make up less than 1% of total abundance: Nitrospirae, Armatimonadetes, Spirochaetes, Synergistetes, Latescibacteria, BRC1, Parcubacteria, Microgenomates, Chlamydiae, Deferribacteres, Fibrobacteres, Candidatus Saccharibacteria, Elusimicrobia, candidate division WPS-2, SR1, Lentisphaerae, Hydrogenedentes, Cloacimonetes, Ignavibacteriae, Deinococcus-Thermus, and Fusobacteria.

Using 16S rRNA gene high-throughput sequencing, the microbial community structure of the samples was characterized (Fig 1). The manure community was unique compared to the soil communities having significantly more members from the Bacteroidetes and Firmicutes phyla than the soil communities (P<0.05), while also having significantly lower percentages of the remaining phyla compared to soil and manure line samples (P<0.05). Although the most abundant phyla stayed fairly consistent in soil samples across our study (Fig 1), we did observe significant changes with specific phyla. Soil prior to manure application had significantly more candidate division WPS-1 OTUs than all of the other soil and manure line samples (P<0.01). After manure application, manure line soil had significantly more Firmicutes than the rest of the soil sample dates (P<0.01). The manure line also had significantly more Proteobacteria than the soil four months after manure application (P<0.05). Fall post-manure soil had significantly more unclassified bacteria than pre-manure, manure line, and spring time 2 soil (P<0.05). The spring soil samples had significantly more Verrucomicrobia than the rest of the soil samples but were not significantly different between each other (P<0.01).

### Bacterial community α-diversity

**Fig 2:**
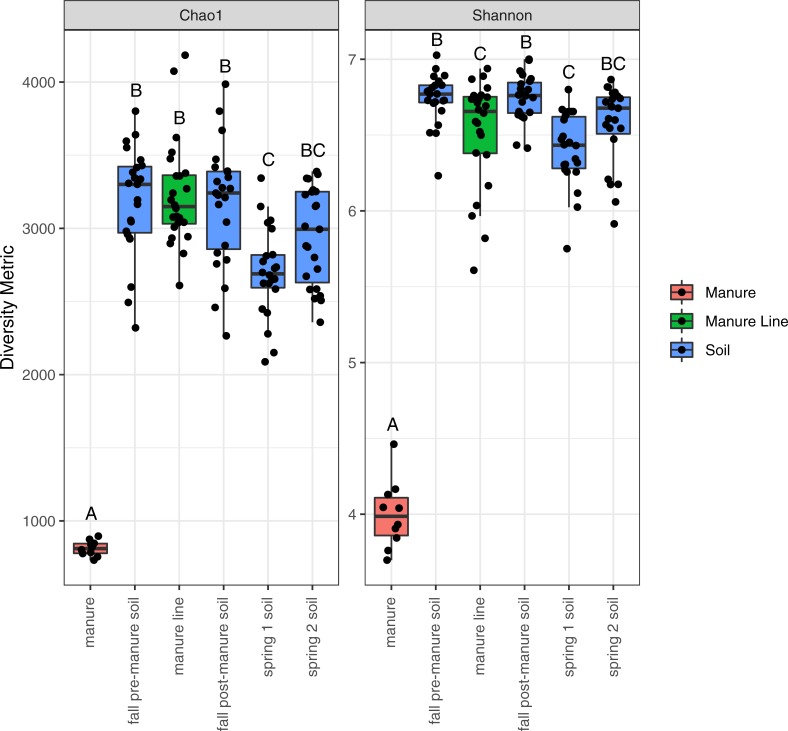
Alpha diversity indices of microbiome samples. Boxplots represent 25th to 75th percentiles and whiskers showing a maximum of 1.5x the interquartile range (IQR). Different letters indicate significant differences within the alpha diversity indexes (P<0.05).

The alpha diversity, the diversity within each sample type, was determined using the Chao1 and Shannon diversity indices (Fig 2). Alpha diversity was significantly lower in the manure compared to the soil and manure line samples. In both the alpha diversity measures, there was an insignificant difference in diversity between fall pre-manure and post-manure soil. There was a significant decrease in diversity between pre-manure and spring time 1 soil, but no significant difference between pre-manure and spring time 2 soil diversity. The manure line alpha diversity was similar to the soil alpha diversity. However, the difference between fall pre-manure soil and manure line diversity was significant using the Shannon index, but not using Chao1.

### Community comparisons (β-diversity)

**Fig 3:**
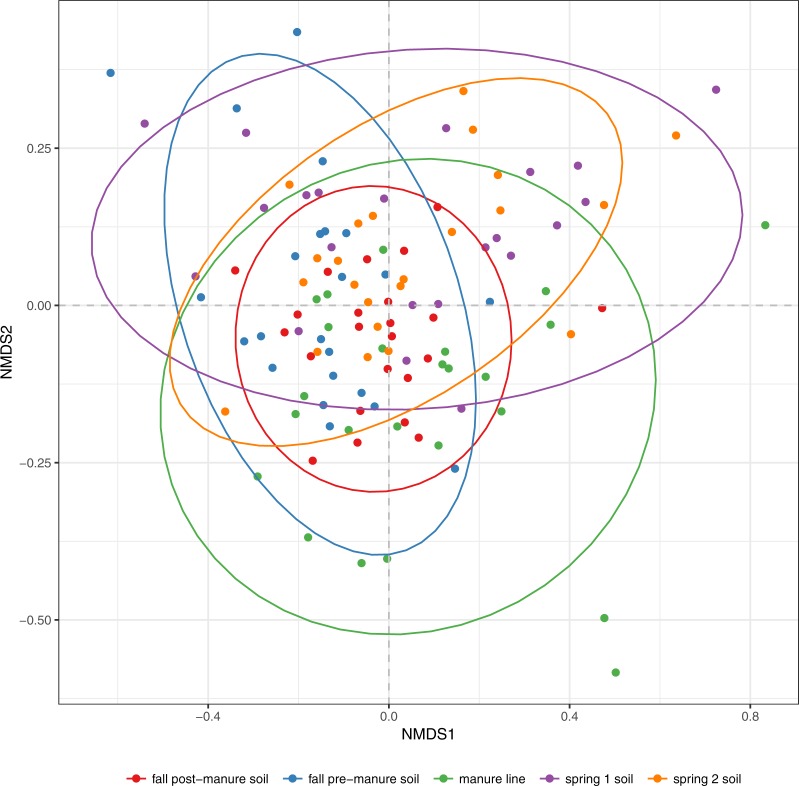
Comparison of community composition between soil and manure line samples. Community composition varied significantly between all samples except between the spring samples. Spring time 1 and spring time 2 soil are combined as spring soil. Clustering is based on nonmetric multidimensional scaling analysis of samples according to Bray-Curtis distances. Manure was excluded from the NMDS plot to show variation of soil samples.

The NMDS ordination and PERMANOVA were utilized to compare the bacterial community between samples. Pairwise PERMANOVA showed that the manure community was significantly different than the soil and manure line samples (P<0.001). While the soil and manure line community compositions shared the same dominant phyla, pairwise PERMANOVA, which considers the whole community composition, showed that the bacterial community differed significantly between all soil and manure line samples, except for the spring samples (P<0.001, Fig 3). Both time and sample type were significant factors in the difference of microbiome composition between soil and manure line samples (P<0.05). Sample date explained 27.6% of the variance while type of sample explained 0.023% of variance.

### Dispersion of manure OTUs

**Fig 4:**
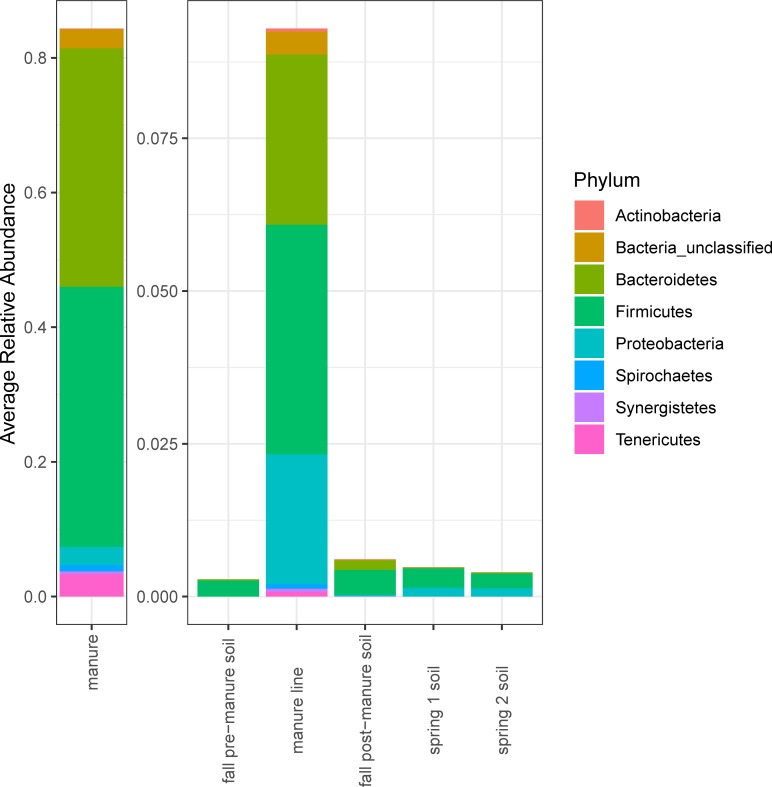
Average relative abundance of manure associated OTUs. OTUs are classified as manure associated if abundances were significantly greater (P<0.05) in manure compared to fall pre-manure soil.

To determine if the changes in the soil community post-manure application were due to the addition of OTUs from manure, 104 OTUs significantly more abundant (P<0.05) in manure than pre-manure soil were identified and tracked (S1 Table). The manure associated OTUs were used as indicators of dispersion from the manure microbiome and their changes in abundance between pre-manure soil and post-manure soil samples were measured. In the manure line samples immediately following manure application, 57 of the 104 identified manure associated OTUs were significantly elevated from pre-manure soil abundance (Fig 4). In the soil samples taken in between injection lines, only seven manure associated OTUs were significantly elevated in the fall post-manure soil from pre-manure soil. In both the spring sampling dates, the same three manure associated OTUs were significantly more abundant than pre-manure soil. Of the three OTUs that remained elevated in the spring, two were of the Proteobacteria phylum and one was of the Firmicutes phylum.

Of the 104 OTUs identified as being significantly more abundant in manure than pre-manure soil, 69 were only present in manure and not in pre-manure soil. These OTUs were also tracked. Thirty-eight of the manure specific OTUs were found in the manure line. Only one manure specific OTU, a Proteobacteria belonging to the genus *Pseudomonas*, remained in the fall post-manure soil. This same OTU was the only OTU present in manure and not pre-manure soil that remained significantly elevated in both spring soil samples. Pseudomonas are known to be well adapted to soil and agricultural environments along with many also living as opportunistic pathogens. We propose this OTU could have the ability to grow and survive in both animal and soil environments.

### Resistance gene and mobile genetic element abundance

**Fig 5:**
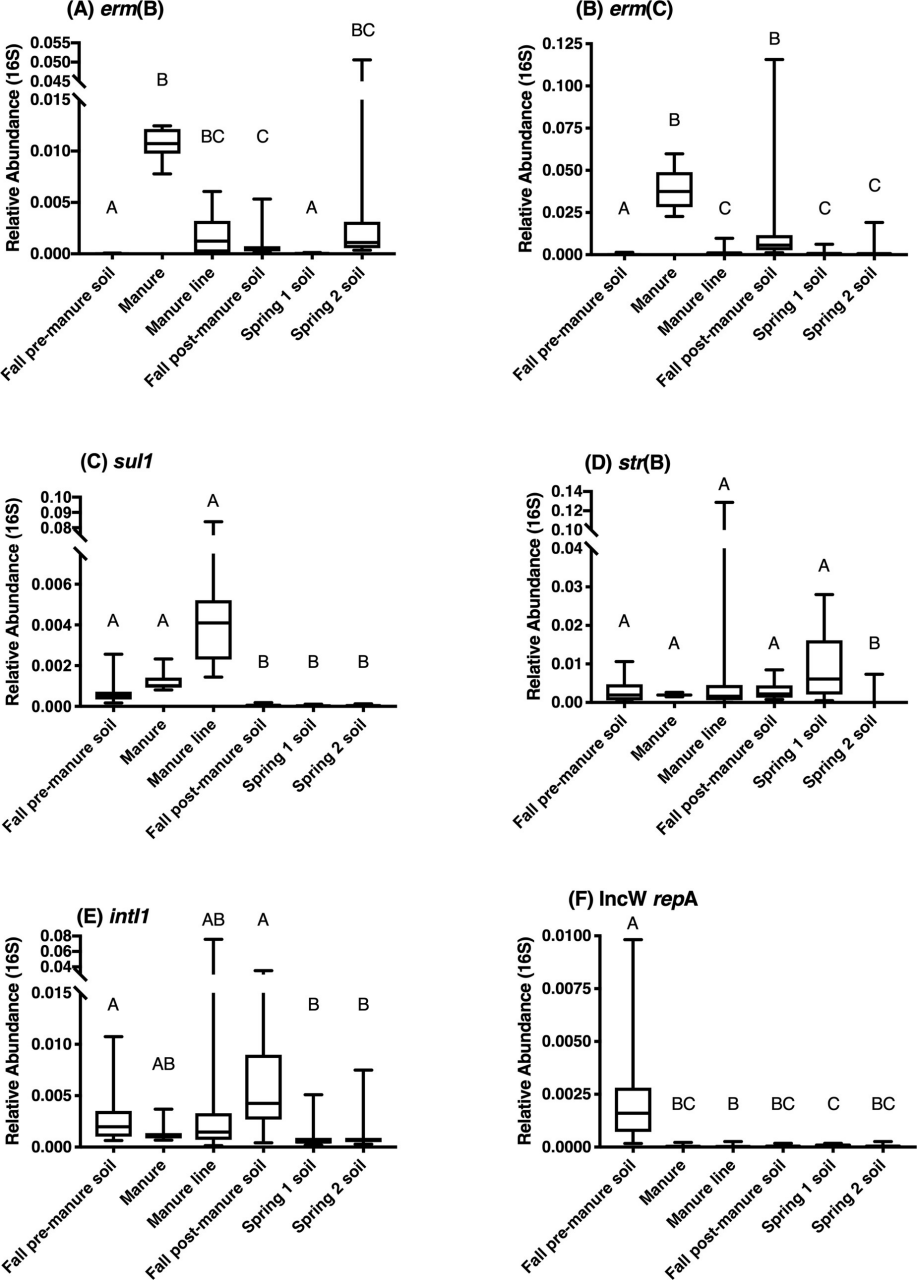
Relative abundance of target genes to 16S rRNA copy number. Different letters indicate significant differences (P<0.05). n = 10 for manure. n = 20 for soil samples.

Along with the community characterization, we investigated if the abundance of antibiotic resistance genes and mobile genetic elements in soil were significantly enriched by manure application, as it is possible that even if manure bacteria do not survive in the soil, they may be able to pass along their ARGs to members of the soil microbiome. Abundances of target ARGs and mobile genetic elements were measured in samples relative to 16S rRNA gene. Every gene was detected in at least one sample of every sample type. Manure generally had the highest abundances of the target genes. However, relative abundances of each gene varied considerably in all samples and the abundance pattern of each gene was unique (Fig 5).

*erm(B)* and *erm(C)* had similar dynamics after manure application, with significantly higher abundance in fall post-manure soil and manure line samples compared to pre-manure soil. *erm(C)* abundances remained significantly higher in both spring sampling dates compared to pre-manure soil. However, *erm(B)* abundance lowered to pre-manure levels in the spring time 1 soil, but abundance was significantly elevated again from pre-manure soil in the spring time 2 soil to a level similar to post-manure soil abundance.

The other gene targets were not significantly increased following manure application (Fig 5). *sul1* abundance decreased in the fall post-manure soil and remained at a lower level throughout the spring samples. Abundance of *str(B)* was similar in all the samples, except there was a significant decrease in the soil three months after manure application where *str(B)* abundance was mostly below the limit of quantification. Like *str(B)*, all the fall soil, manure, and manure line samples had similar abundances of *intI1*. However, there was a significant decrease in abundance of *intI1* in both the spring sample dates. IncW *rep*A abundance significantly dropped in soil and manure line after manure application and stayed significantly lowered throughout the spring.

## Discussion

Utilizing high throughput sequencing and quantitative PCR, this study characterized changes in the microbial community and resistance gene abundance following the application of swine manure in the soil of a commercial Iowa farm. The soil microbiome significantly changed throughout the experiment. However, the addition of the manure microbiome likely had limited influence on soil community composition as the microbes in the manure had short persistence in the soil. A similar trend was observed for ARGs as the target antibiotic resistance and mobile genetic element genes did not display uniform dynamics in response to manure application. These results suggest the response of the soil microbiome to manure amendment is complex and dependent on many environmental factors.

### Soil microbiome dynamics

High throughput 16S rRNA sequencing was employed to characterize the bacterial microbiome of manure and of the farmland prior to and following manure application. The dominant phyla of both the manure and soil microbiomes in this study were generally characteristic of microbiomes described in prior studies [6, 9, 21, 51–54]. Both the soil alpha diversity and phyla composition were fairly consistent across all sampling dates. Unlike our results, some studies have found that manure amendment increases soil diversity and significantly changes the abundance of the major phyla after manure application [55, 56]. Other studies have found no change in alpha diversity and phyla composition of the bacterial community after manure application [57, 58]. Sample date was a significant factor in microbiome composition suggesting that the microbiome composition changes throughout time.

Our results are comparable to numerous other studies which have found manure application to significantly alter the microbe community in soils [9, 59–61]. Studies that have examined the soil community response after manure application longitudinally have found results similar to the present study in that the soil microbiome shifted away from the community prior to manure application [61, 62]. Two long-term studies found that 40 years of annual manure application significantly altered the soil microbiome compared to control soil [14, 63]. The soil microbiome composition and function have been shown to be very sensitive and, in a majority of studies, never fully recovers from disturbances [64, 65]. These differing results suggest that the response of the soil microbiome to manure amendment may depend on factors like regional soil characteristics and weather [66]. Our study was conducted on farm soil just before and following seasonal frost, ice and snow, which likely impacted dispersion of microbes and microbial survivorship over a three-month winter period.

The significant differences of the bacterial community between soil before and after manure application and between manure line and soil suggest that the introduction of the manure microbes could be a source of the change. To determine the impact of the addition of the manure microbes on the soil microbiome, OTUs significantly more abundant in the manure microbiome compared to the pre-manured soil were measured in post-manure soil samples. About half of the manure associated OTUs, most of which were present in manure and absent in pre-manure soil, were significantly elevated in the manure line samples suggesting that part of the soil microbiome shift is due to the addition of bacteria originating from manure. However, almost all of the manure associated OTUs returned to pre-manure levels of abundance by the spring which suggests that the influence of dispersion originating from the manure microbiome is temporary. The fall post-manure soil samples, which had low levels of manure associated OTUS, were taken between injection lines and thus were not in direct contact with manure. Nutrient addition from manure application may be another major factor in shifting the soil microbiome [67], but the limited duration of the manure associated bacteria in the soil combined with the fact that there was no pattern in the phyla abundance changes suggests that it is unlikely that manure was the sole driving factor in the soil microbiome shifts throughout the time points measured. Analysis at the family level follows what was observed at the phyla level with no clear patterns beyond the significant differences between soil and manure (S1 Fig). Previous studies have found that manure-associated bacteria had only a temporary effect on soil the microbiome and suggested that most manure-associated bacteria are not well adapted to survive in soil [68, 69]. In a microcosm study, Rieke et al. found that most manure associated OTUs which elevated in soil after manure application began to decrease in soil after 24 days [70]. However, it is possible that the manure microbiome is instead dispersing to elsewhere in the environment. Other factors like temperature, soil moisture, and soil pH can also have significant effects on microbiomes [6, 71]. Future research should determine the environmental characteristics that contribute to the composition of the soil microbiome.

### Dynamics of ARGs and mobile genetic elements abundance

Abundance of select antibiotic resistance and mobile element target genes were measured to determine their dynamics in soil after manure application. Gene target abundances in almost all the soil samples were low. Within samples ARG abundances varied considerably, which is consistent with the heterogeneous nature of soil and previous studies [6, 72, 73]. Each gene displayed a unique abundance pattern across time points. An increase in gene abundance can be either from the direct addition of bacteria originating from manure, from the proliferation of bacteria already in the soil, or from the spread of genes due to horizontal gene transfer [23, 74]. Likewise, a decrease in gene abundance may be attributed to either the natural decay of the genes or spread of genes to other parts of the environment due to factors such as precipitation and temperature [4, 25, 75–81]. A recent study by Wang and colleagues, determined that after 26 years of manure application, there was not a large accumulation of ARGs in the soils studied and the low fold increases found in ARGs were not consistent across all types of ARGs [66].

The antibiotic resistant gene abundance patterns in the present study may be connected to the previous antibiotic exposure of the hogs. The abundance of *erm(B)*, *erm(C*), and *sul1* all increased after manure application. The manure producing hogs were given sulfamethazine, which *sul1* provides resistance, and the macrolide tylosin phosphate, which both *erm(B)* and *erm(C)* may provide cross resistance [82, 83]. Abundance of *sul1* has also been previously shown to be positively correlated with the amount of sulfonamide in soil [84]. The hogs were not given any streptomycin or other aminoglycosides of which *str(B)* provides resistance to [85]. It is important to note that *str(B)* was still detected in all samples. This may mean the *str(B)* abundances may be a result of naturally occurring antibiotics present in the soil or from a previous course of antibiotics, as resistance is known to persist for years after antibiotics have been administered [86, 87]. However, Udikovic-Kolic et al. found that cow manure amendment increased ARG abundance independent of the previous antibiotic exposure of the manure producing cows suggesting that ARGs may be enriched rather than introduced from manure application [22]. It is unknown whether the changes in gene abundance in this study were directly due to the addition of manure or due to other environmental factors. The extensive use of manure fertilizer along with grazing animals made us unable to obtain control soil that had no animal impact.

ARGs are often found to be linked to mobile element genes in the environment. The genes *intI1*, an integrase gene which allows exogenous genes to be inserted in the genome [88], and IncW *repA*, which assists in replication of plasmids carrying resistance genes [89], did not increase in abundance after manure application in our study. However, other studies have found both ARGs and mobile element genes to be increased following manure application [90, 91]. Recent work by Zhao and colleagues has found a link between the presence of metals in soils and ARGs and mobile element gene selection, a new factor to consider for horizontal gene transfer [92].

To our knowledge, the present field study is the first of its kind to examine soil after manure application from a swine CAFO in Iowa, but many studies have shown trends of increase in ARG abundance in soil after manure application [23, 24, 90, 91, 93, 94]. Marti et al. measured the abundance of five of the six genes in the present study in an agricultural field for 304 days after manure application and compared abundance to soil that had no manure exposure [23]. Their measured abundance dynamics of *erm(B)*, *str(B)*, and IncW *repA* in manure applied soil were similar to the present study and the abundances of the genes were significantly elevated from control soil [23]. Unlike this study, the abundance of *sul1* and *intI1* in Marti et al. both seemed to be significantly increased by manure application [23]. The differences observed across studies are likely due to many confounding factors including differences in antibiotics given to the animals that produced the manure, water availability, bacterial community composition, and soil pH.

The present study found that the fate of gene abundances depends on the gene. Two genes, *erm(B)* and *erm(C)*, were significantly elevated in abundance in the spring 2 sampling. *erm(B)* remained elevated through all time points, while *erm(C*) was below quantification at spring time 1 and returned to elevated levels at spring time 2. The other four target genes were either at a similar or a lower abundance than pre-manure soil. Marti et al., which followed five of the genes in this study, suggests an offset time of at least one growing period is necessary to allow abundance to safely return to pre-application conditions [23]. In another study, soils treated with swine manure returned to ARG levels in untreated soil within 20 days [95]. However, another study found significantly elevated levels of ARGs in soil after 25 years of swine manure application [58], which can be contrasted with Wang et al. who found relatively low fold changes after 26 years of manure application [66]. Studies have shown that gene abundances are sensitive to factors like soil type [96], application type [97], and the presence of plants [98]. Further studies are needed to better characterize the fate and dynamics of ARGs following manure application, especially because the results of this study suggest that abundance generalizations cannot be made across genes and ARGs have been shown to have different rates of dissipation [73]. Additionally, future research may be able to link certain resistance genes to specific taxa in the environment.

## Supporting information

S1 Fig Abundance of the ten most abundant families within each sample. Different letters indicated significant differences within each family by ANOVA with Tukey post-hoc test (P<0.05).
(DOCX)

S1 Table Relative abundance of manure associated OTUs. (DOCX)
