# Supplementary material for: Characterizing the soil microbiome and quantifying antibiotic resistance gene dynamics in agricultural soil following swine CAFO manure application
Source: PLoS One. 2019 Aug 19;14(8):e0220770. doi: 10.1371/journal.pone.0220770 (PMC6699696; doi:10.1371/journal.pone.0220770)
Supplement: S1 Fig — Different letters indicated significant differences within each family by ANOVA with Tukey post-hoc test (P<0.05). (DOCX) [file pone.0220770.s001.docx]

B

BC

C

B

B

A

A

B

C

C

B

B

C

B

A

BC

BC

C

B

B

C

B

B

BC

A

B

B

B

B

A

B

B

B

A

BC

C

BC

B

A

BC

C

B

A

C

C

BC

BC

A

BC

BC

BC

C

A

B

DC

BC

B

C

D

B
