# Supplementary material for: Characterizing the soil microbiome and quantifying antibiotic resistance gene dynamics in agricultural soil following swine CAFO manure application
Source: PLoS One. 2019 Aug 19;14(8):e0220770. doi: 10.1371/journal.pone.0220770 (PMC6699696; doi:10.1371/journal.pone.0220770)
Supplement: S1 Table — (DOCX) [file pone.0220770.s002.docx]

| **OTU** | **Phylum** | **Genus** | **Manure** | **Fall pre-manure soil** | **Manure line** | **Fall post-manure soil** | **Spring 1 soil** | **Spring 2 soil** |
| --- | --- | --- | --- | --- | --- | --- | --- | --- |
| 2 | Bacteroidetes | Bacteroidales_unclassified | 1.40E-01 | 4.97E-05 | 1.65E-02 | 9.85E-04 | 4.25E-05 | 4.33E-05 |
| 5 | Bacteroidetes | Petrimonas | 1.23E-01 | 5.42E-05 | 6.28E-03 | 2.37E-04 | 4.02E-05 | 2.17E-05 |
| 7 | Firmicutes | Clostridiales_Incertae_Sedis_XI_unclas. | 8.34E-02 | 2.49E-05 | 1.33E-02 | 9.40E-04 | 1.65E-05 | 8.66E-06 |
| 11 | Firmicutes | Clostridium_sensu_stricto | 8.43E-02 | 1.35E-03 | 4.23E-03 | 9.74E-04 | 1.43E-03 | 1.17E-03 |
| 21 | Firmicutes | Firmicutes_unclassified | 4.46E-02 | 4.52E-06 | 4.73E-03 | 3.57E-04 | 1.13E-04 | 9.31E-05 |
| 39 | Bacteroidetes | Bacteroidales_unclassified | 2.75E-02 | 4.52E-06 | 2.68E-03 | 1.85E-04 | 2.84E-05 | 2.17E-06 |
| 51 | Tenericutes | Acholeplasma | 2.50E-02 | 0.00E+00 | 5.09E-04 | 1.13E-05 | 2.36E-06 | 8.66E-06 |
| 60 | Proteobacteria | Pseudomonadaceae_unclassified | 5.46E-04 | 4.52E-06 | 8.82E-03 | 5.88E-05 | 2.55E-04 | 8.23E-05 |
| 75 | Proteobacteria | Pseudomonas | 6.76E-04 | 4.52E-06 | 7.77E-03 | 4.75E-05 | 7.32E-05 | 4.55E-05 |
| 86 | Bacteria_unclassified | Bacteria_unclassified | 1.55E-02 | 0.00E+00 | 1.10E-03 | 6.78E-06 | 2.36E-06 | 4.33E-06 |
| 94 | Firmicutes | Clostridium_sensu_stricto | 1.29E-02 | 3.28E-04 | 9.49E-04 | 2.19E-04 | 3.78E-04 | 2.12E-04 |
| 102 | Firmicutes | Terrisporobacter | 1.20E-02 | 3.25E-04 | 1.11E-03 | 1.92E-04 | 4.02E-04 | 2.53E-04 |
| 123 | Firmicutes | Firmicutes_unclassified | 1.02E-02 | 4.52E-06 | 1.46E-03 | 2.01E-04 | 2.36E-05 | 2.82E-05 |
| 138 | Proteobacteria | Pseudomonas | 4.94E-04 | 0.00E+00 | 2.43E-03 | 2.26E-06 | 1.10E-03 | 1.20E-03 |
| 149 | Firmicutes | Phascolarctobacterium | 9.55E-03 | 1.36E-05 | 7.23E-04 | 1.18E-04 | 9.45E-06 | 2.38E-05 |
| 166 | Firmicutes | Bacillales_unclassified | 1.21E-02 | 9.04E-06 | 6.93E-05 | 1.13E-05 | 0.00E+00 | 0.00E+00 |
| 179 | Bacteroidetes | Bacteroidales_unclassified | 9.39E-03 | 0.00E+00 | 3.34E-04 | 1.58E-05 | 0.00E+00 | 0.00E+00 |
| 187 | Firmicutes | Streptococcus | 1.16E-02 | 0.00E+00 | 3.05E-04 | 2.71E-05 | 1.65E-05 | 4.33E-06 |
| 193 | Bacteroidetes | Bacteroides | 8.36E-03 | 0.00E+00 | 4.85E-04 | 1.81E-05 | 0.00E+00 | 4.33E-06 |
| 202 | Firmicutes | Turicibacter | 7.70E-03 | 1.74E-04 | 4.74E-04 | 1.33E-04 | 1.82E-04 | 9.75E-05 |
| 213 | Firmicutes | Romboutsia | 6.52E-03 | 1.74E-04 | 6.15E-04 | 1.69E-04 | 2.39E-04 | 1.82E-04 |
| 214 | Firmicutes | Clostridium_sensu_stricto | 6.35E-03 | 2.87E-04 | 6.22E-04 | 1.40E-04 | 2.08E-04 | 1.78E-04 |
| 219 | Proteobacteria | Marinospirillum | 1.02E-02 | 0.00E+00 | 6.71E-05 | 4.52E-06 | 0.00E+00 | 0.00E+00 |
| 236 | Bacteroidetes | Prevotella | 8.09E-03 | 3.16E-05 | 7.58E-05 | 5.42E-05 | 4.73E-06 | 7.36E-05 |
| 254 | Bacteria_unclassified | Bacteria_unclassified | 7.98E-03 | 0.00E+00 | 3.25E-05 | 0.00E+00 | 0.00E+00 | 0.00E+00 |
| 286 | Firmicutes | Syntrophaceticus | 3.00E-03 | 4.52E-06 | 1.41E-03 | 1.94E-04 | 0.00E+00 | 1.08E-05 |
| 299 | Spirochaetes | Treponema | 4.80E-03 | 0.00E+00 | 6.26E-04 | 4.29E-05 | 2.13E-05 | 6.50E-06 |
| 308 | Proteobacteria | Xanthomonadaceae_unclassified | 3.13E-03 | 4.52E-06 | 1.19E-03 | 7.23E-05 | 2.36E-06 | 0.00E+00 |
| 325 | Firmicutes | Ruminococcaceae_unclassified | 5.05E-03 | 0.00E+00 | 6.61E-04 | 3.16E-05 | 0.00E+00 | 0.00E+00 |
| 327 | Bacteroidetes | Porphyromonadaceae_unclassified | 5.71E-03 | 0.00E+00 | 3.70E-04 | 2.03E-05 | 0.00E+00 | 0.00E+00 |
| 332 | Bacteroidetes | Anaerocella | 6.53E-03 | 0.00E+00 | 1.02E-04 | 1.81E-05 | 0.00E+00 | 6.50E-06 |
| 344 | Tenericutes | Acholeplasma | 5.96E-03 | 2.26E-06 | 2.10E-04 | 2.26E-06 | 0.00E+00 | 0.00E+00 |
| 346 | Bacteria_unclassified | Bacteria_unclassified | 1.27E-03 | 9.04E-06 | 1.84E-03 | 8.14E-05 | 4.73E-06 | 1.08E-05 |
| 397 | Firmicutes | Enterococcaceae_unclassified | 3.26E-03 | 1.58E-05 | 6.09E-04 | 9.27E-05 | 5.91E-05 | 1.52E-05 |
| 399 | Firmicutes | Clostridiales_unclassified | 5.19E-03 | 0.00E+00 | 1.04E-04 | 0.00E+00 | 0.00E+00 | 2.17E-06 |
| 426 | Bacteroidetes | Bacteroidales_unclassified | 4.44E-03 | 0.00E+00 | 1.97E-04 | 2.03E-05 | 0.00E+00 | 2.17E-06 |
| 431 | Bacteroidetes | Marinilabiliaceae_unclassified | 4.24E-03 | 0.00E+00 | 2.71E-04 | 9.04E-06 | 0.00E+00 | 0.00E+00 |
| 448 | Proteobacteria | Proteobacteria_unclassified | 4.40E-03 | 0.00E+00 | 9.53E-05 | 2.26E-06 | 0.00E+00 | 0.00E+00 |
| 457 | Firmicutes | Atopostipes | 2.35E-03 | 0.00E+00 | 8.94E-04 | 8.59E-05 | 3.78E-05 | 2.17E-06 |
| 461 | Firmicutes | Sporanaerobacter | 2.73E-03 | 6.78E-06 | 6.67E-04 | 2.26E-05 | 0.00E+00 | 4.33E-06 |
| 475 | Firmicutes | Firmicutes_unclassified | 3.96E-03 | 0.00E+00 | 1.32E-04 | 6.78E-06 | 2.36E-06 | 4.33E-06 |
| 498 | Firmicutes | Megasphaera | 4.26E-03 | 5.88E-05 | 2.38E-05 | 2.71E-05 | 1.42E-05 | 4.11E-05 |
| 503 | Synergistetes | Aminobacterium | 2.78E-03 | 0.00E+00 | 4.61E-04 | 3.39E-05 | 0.00E+00 | 0.00E+00 |
| 542 | Firmicutes | Ruminococcaceae_unclassified | 3.21E-03 | 4.52E-06 | 1.93E-04 | 9.04E-06 | 2.36E-06 | 4.33E-06 |
| 547 | Firmicutes | Clostridiales_unclassified | 2.99E-03 | 0.00E+00 | 3.68E-04 | 3.84E-05 | 1.42E-05 | 4.33E-06 |
| 556 | Bacteroidetes | Prevotella | 4.54E-03 | 2.26E-05 | 2.82E-05 | 6.78E-06 | 2.36E-06 | 4.33E-06 |
| 591 | Firmicutes | Anaerococcus | 3.41E-03 | 4.52E-06 | 9.31E-05 | 0.00E+00 | 0.00E+00 | 0.00E+00 |
| 592 | Firmicutes | Ruminococcaceae_unclassified | 2.87E-03 | 0.00E+00 | 1.75E-04 | 1.58E-05 | 0.00E+00 | 2.17E-06 |
| 593 | Spirochaetes | Sphaerochaeta | 3.09E-03 | 0.00E+00 | 5.41E-05 | 0.00E+00 | 0.00E+00 | 0.00E+00 |
| 596 | Firmicutes | Selenomonas | 3.98E-03 | 2.26E-06 | 4.76E-05 | 1.58E-05 | 1.18E-05 | 1.30E-05 |
| 612 | Firmicutes | Clostridiales_Incertae_Sedis_XI_unclas. | 3.30E-03 | 0.00E+00 | 6.93E-05 | 2.26E-06 | 0.00E+00 | 8.66E-06 |
| 673 | Firmicutes | Ruminococcaceae_unclassified | 2.71E-03 | 0.00E+00 | 1.02E-04 | 6.78E-06 | 2.36E-06 | 0.00E+00 |
| 707 | Firmicutes | Lachnospiraceae_unclassified | 1.23E-03 | 2.26E-06 | 6.13E-04 | 3.84E-05 | 0.00E+00 | 0.00E+00 |
| 710 | Bacteroidetes | Bacteroides | 2.45E-03 | 0.00E+00 | 1.52E-04 | 1.36E-05 | 4.73E-06 | 0.00E+00 |
| 748 | Firmicutes | Clostridiales_Incertae_Sedis_XI_unclas. | 1.07E-03 | 0.00E+00 | 5.89E-04 | 4.75E-05 | 1.18E-05 | 0.00E+00 |
| 768 | Proteobacteria | Ignatzschineria | 2.03E-03 | 0.00E+00 | 2.23E-04 | 1.58E-05 | 0.00E+00 | 0.00E+00 |
| 776 | Tenericutes | Acholeplasma | 2.59E-03 | 0.00E+00 | 6.93E-05 | 0.00E+00 | 0.00E+00 | 0.00E+00 |
| 800 | Firmicutes | Ruminococcaceae_unclassified | 2.49E-03 | 6.78E-06 | 7.36E-05 | 2.26E-06 | 4.73E-06 | 1.08E-05 |
| 805 | Firmicutes | Lactobacillus | 1.74E-03 | 9.04E-06 | 1.65E-04 | 3.16E-05 | 2.84E-05 | 2.17E-05 |
| 826 | Bacteroidetes | Bacteroides | 2.25E-03 | 0.00E+00 | 1.02E-04 | 6.78E-06 | 0.00E+00 | 0.00E+00 |
| 842 | Proteobacteria | Succinivibrio | 1.95E-03 | 0.00E+00 | 6.93E-05 | 1.13E-05 | 0.00E+00 | 0.00E+00 |
| 868 | Firmicutes | Ruminococcaceae_unclassified | 2.12E-03 | 0.00E+00 | 7.36E-05 | 0.00E+00 | 0.00E+00 | 0.00E+00 |
| 878 | Firmicutes | Ruminococcaceae_unclassified | 1.90E-03 | 0.00E+00 | 7.15E-05 | 6.78E-06 | 0.00E+00 | 0.00E+00 |
| 883 | Firmicutes | Ruminococcaceae_unclassified | 1.83E-03 | 0.00E+00 | 1.10E-04 | 1.13E-05 | 0.00E+00 | 8.66E-06 |
| 918 | Bacteroidetes | Bacteroidetes_unclassified | 2.08E-03 | 1.58E-05 | 5.20E-05 | 2.26E-06 | 2.36E-06 | 4.33E-06 |
| 956 | Firmicutes | Lachnospiraceae_unclassified | 1.24E-03 | 0.00E+00 | 2.77E-04 | 1.81E-05 | 0.00E+00 | 8.66E-06 |
| 958 | Firmicutes | Veillonellaceae_unclassified | 2.30E-03 | 6.78E-06 | 3.47E-05 | 1.13E-05 | 0.00E+00 | 8.66E-06 |
| 973 | Bacteria_unclassified | Bacteria_unclassified | 1.34E-03 | 0.00E+00 | 2.27E-04 | 1.81E-05 | 7.09E-06 | 0.00E+00 |
| 1028 | Bacteroidetes | Flavobacteriaceae_unclassified | 1.57E-03 | 0.00E+00 | 7.15E-05 | 0.00E+00 | 0.00E+00 | 0.00E+00 |
| 1030 | Firmicutes | Firmicutes_unclassified | 1.43E-03 | 2.26E-06 | 1.71E-04 | 9.04E-06 | 0.00E+00 | 2.38E-05 |
| 1086 | Firmicutes | Bacillales_unclassified | 1.70E-03 | 0.00E+00 | 7.36E-05 | 9.04E-06 | 0.00E+00 | 0.00E+00 |
| 1144 | Bacteroidetes | Bacteroidetes_unclassified | 1.47E-03 | 2.26E-06 | 3.90E-05 | 2.26E-06 | 0.00E+00 | 0.00E+00 |
| 1148 | Bacteria_unclassified | Bacteria_unclassified | 1.02E-03 | 0.00E+00 | 2.12E-04 | 6.78E-06 | 0.00E+00 | 0.00E+00 |
| 1149 | Proteobacteria | Succinivibrio | 1.22E-03 | 0.00E+00 | 1.41E-04 | 0.00E+00 | 0.00E+00 | 0.00E+00 |
| 1178 | Spirochaetes | Treponema | 1.39E-03 | 0.00E+00 | 7.15E-05 | 0.00E+00 | 0.00E+00 | 1.08E-05 |
| 1192 | Proteobacteria | Oligella | 4.99E-04 | 0.00E+00 | 3.44E-04 | 1.58E-05 | 2.36E-06 | 0.00E+00 |
| 1200 | Firmicutes | Lachnospiraceae_unclassified | 1.47E-03 | 0.00E+00 | 6.28E-05 | 1.13E-05 | 0.00E+00 | 0.00E+00 |
| 1202 | Firmicutes | Lachnospiraceae_unclassified | 1.13E-03 | 0.00E+00 | 1.56E-04 | 2.26E-06 | 0.00E+00 | 0.00E+00 |
| 1230 | Firmicutes | Coprococcus | 1.20E-03 | 0.00E+00 | 1.08E-04 | 6.78E-06 | 0.00E+00 | 0.00E+00 |
| 1260 | Actinobacteria | Corynebacterium | 4.00E-04 | 0.00E+00 | 3.05E-04 | 2.94E-05 | 2.36E-05 | 1.08E-05 |
| 1306 | Firmicutes | Clostridiales_Incertae_Sedis_XI_unclas. | 6.13E-04 | 0.00E+00 | 2.32E-04 | 1.81E-05 | 0.00E+00 | 0.00E+00 |
| 1309 | Firmicutes | Peptostreptococcaceae_unclassified | 1.12E-03 | 4.52E-06 | 6.50E-05 | 9.04E-06 | 9.45E-06 | 1.08E-05 |
| 1326 | Synergistetes | Synergistaceae_unclassified | 1.16E-03 | 0.00E+00 | 4.55E-05 | 2.26E-06 | 0.00E+00 | 0.00E+00 |
| 1384 | Firmicutes | Clostridiales_unclassified | 1.10E-03 | 0.00E+00 | 3.90E-05 | 9.04E-06 | 2.36E-06 | 2.17E-06 |
| 1403 | Firmicutes | Clostridiales_unclassified | 1.07E-03 | 0.00E+00 | 4.55E-05 | 4.52E-06 | 0.00E+00 | 0.00E+00 |
| 1421 | Bacteria_unclassified | Bacteria_unclassified | 6.24E-04 | 2.26E-06 | 1.99E-04 | 1.13E-05 | 0.00E+00 | 0.00E+00 |
| 1435 | Firmicutes | Lachnospiraceae_unclassified | 7.22E-04 | 4.52E-06 | 1.47E-04 | 6.78E-06 | 2.36E-06 | 0.00E+00 |
| 1440 | Bacteroidetes | Petrimonas | 7.38E-04 | 0.00E+00 | 1.73E-04 | 2.26E-06 | 0.00E+00 | 0.00E+00 |
| 1446 | Firmicutes | Firmicutes_unclassified | 9.82E-04 | 2.26E-06 | 4.98E-05 | 4.52E-06 | 0.00E+00 | 0.00E+00 |
| 1458 | Proteobacteria | Desulfovibrio | 1.21E-03 | 0.00E+00 | 3.03E-05 | 4.52E-06 | 0.00E+00 | 0.00E+00 |
| 1500 | Actinobacteria | Corynebacterium | 4.21E-04 | 0.00E+00 | 1.95E-04 | 3.84E-05 | 4.02E-05 | 6.50E-06 |
| 1545 | Firmicutes | Ruminococcus | 9.51E-04 | 0.00E+00 | 6.06E-05 | 4.52E-06 | 0.00E+00 | 8.66E-06 |
| 1554 | Bacteroidetes | Prevotella | 5.30E-04 | 6.78E-06 | 1.95E-05 | 2.26E-06 | 1.18E-05 | 1.95E-05 |
| 1596 | Firmicutes | Firmicutes_unclassified | 7.28E-04 | 0.00E+00 | 1.23E-04 | 4.52E-06 | 0.00E+00 | 0.00E+00 |
| 1619 | Bacteroidetes | Porphyromonadaceae_unclassified | 1.03E-03 | 0.00E+00 | 2.82E-05 | 0.00E+00 | 0.00E+00 | 0.00E+00 |
| 1626 | Firmicutes | Tissierella | 4.21E-04 | 0.00E+00 | 1.62E-04 | 2.26E-06 | 0.00E+00 | 0.00E+00 |
| 1635 | Firmicutes | Clostridium_sensu_stricto | 6.71E-04 | 3.84E-05 | 3.68E-05 | 4.52E-06 | 3.78E-05 | 5.41E-05 |
| 1647 | Firmicutes | Clostridiales_unclassified | 9.20E-04 | 0.00E+00 | 6.28E-05 | 4.52E-06 | 0.00E+00 | 2.17E-06 |
| 1654 | Firmicutes | Facklamia | 9.88E-04 | 0.00E+00 | 3.25E-05 | 4.52E-06 | 0.00E+00 | 2.17E-06 |
| 1708 | Bacteria_unclassified | Bacteria_unclassified | 7.07E-04 | 0.00E+00 | 1.15E-04 | 0.00E+00 | 0.00E+00 | 0.00E+00 |
| 1838 | Firmicutes | Clostridiales_unclassified | 7.64E-04 | 0.00E+00 | 3.90E-05 | 4.52E-06 | 0.00E+00 | 0.00E+00 |
| 1851 | Firmicutes | Aerococcus | 7.64E-04 | 0.00E+00 | 4.11E-05 | 9.04E-06 | 0.00E+00 | 0.00E+00 |
| 1887 | Bacteroidetes | Bacteroidales_unclassified | 6.97E-04 | 0.00E+00 | 3.68E-05 | 1.36E-05 | 0.00E+00 | 4.33E-06 |
| 1922 | Proteobacteria | Campylobacter | 6.45E-04 | 4.52E-06 | 4.55E-05 | 0.00E+00 | 4.73E-06 | 4.33E-06 |
